# Supplementary material for: A systematic comparison of VBM pipelines and their application to age prediction
Source: Neuroimage. Author manuscript; Available in PMC 2023 Oct 1. (PMC10529438; doi:10.1016/j.neuroimage.2023.120292)
Supplement: 1 [file NIHMS1929002-supplement-1.pdf]

Supplementary Material

- MRI acquisition details

Sample	Scanner	Sequence	Tesla	Slices	Voxel size (mm)	Time parameters (TR / TE / TI [ms])	Other parameters (FA/FOV [^{o}/mm])
eNKI	Siemens Magnetom TrioTim	3D MP-RAGE	3T	176	1 x 1 x 1	1900/2.52/900	9/250x250
Cam-CAN	Tim Trio Siemens	3D MP-RAGE	3.0T	192	1 x 1 x 1	2,250/2.99/900	9/256 x 256
IXI-Guys	Phillips	3D MP-RAGE	1.5T	192	1.2 x 0.94 x 0.94	9.813/4.603/	8/256 x 256
IXI-HH	Discovery GE	3D FSPGR	3T	176	1.2 x 0.94 x 0.94	9.6/4.6/450	N/A /256 x 256
IXI-IOP	GE	N/A	1.5	N/A	1.2 x 0.94 x 0.94	N/A	N/A /256 x 256

Table S.1: MRI acquisition details for the three datasets.

- Preprocessing

All pipelines were run in a high-throughput compute cluster except CAT12.8, which was run in a high-performance computing cluster.

- Study specific templates of each pipeline

The templates created by CAT and ANTs appear to be sharper than those created by FS-LVBM. For ANTs, the template was 197x233x189; for CAT 175x199x175; and for FSL-based pipelines, 91x109x91. Templates built with eNKI are presented in Figure S.1, templates built based on CamCAN subjects can be found in Figure S.2 and for IXI in Figure S.3.

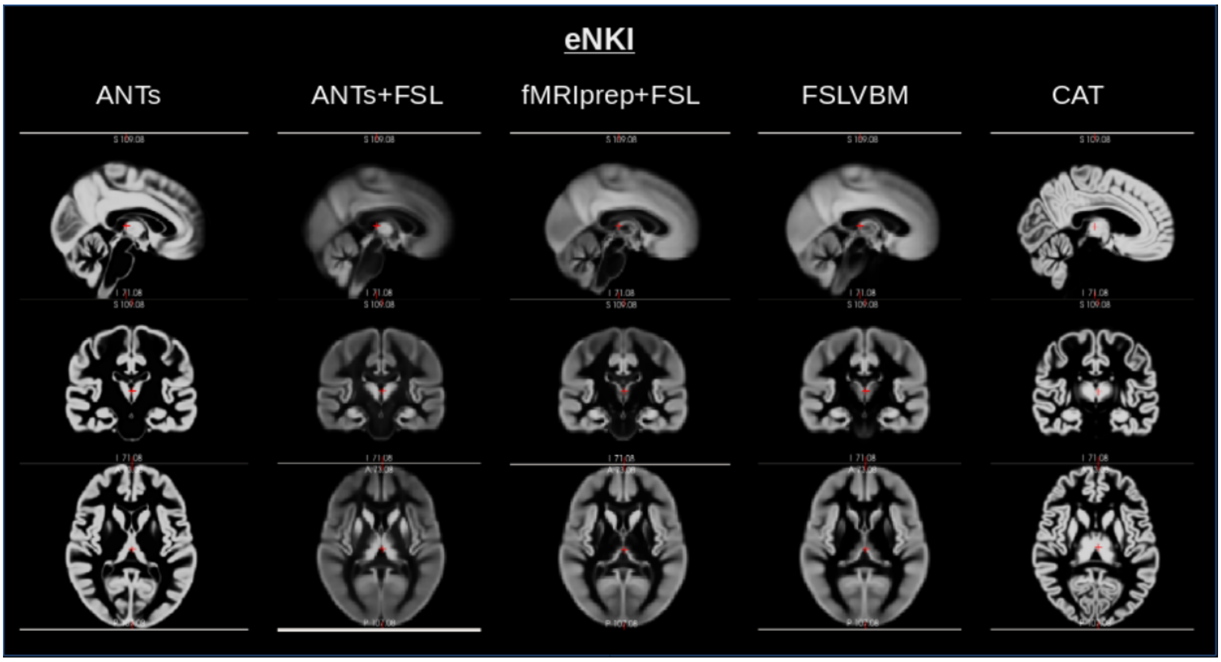

Figure S.1: Templates created for each pipeline. Default CAT and ANTs processes created sharper templates.

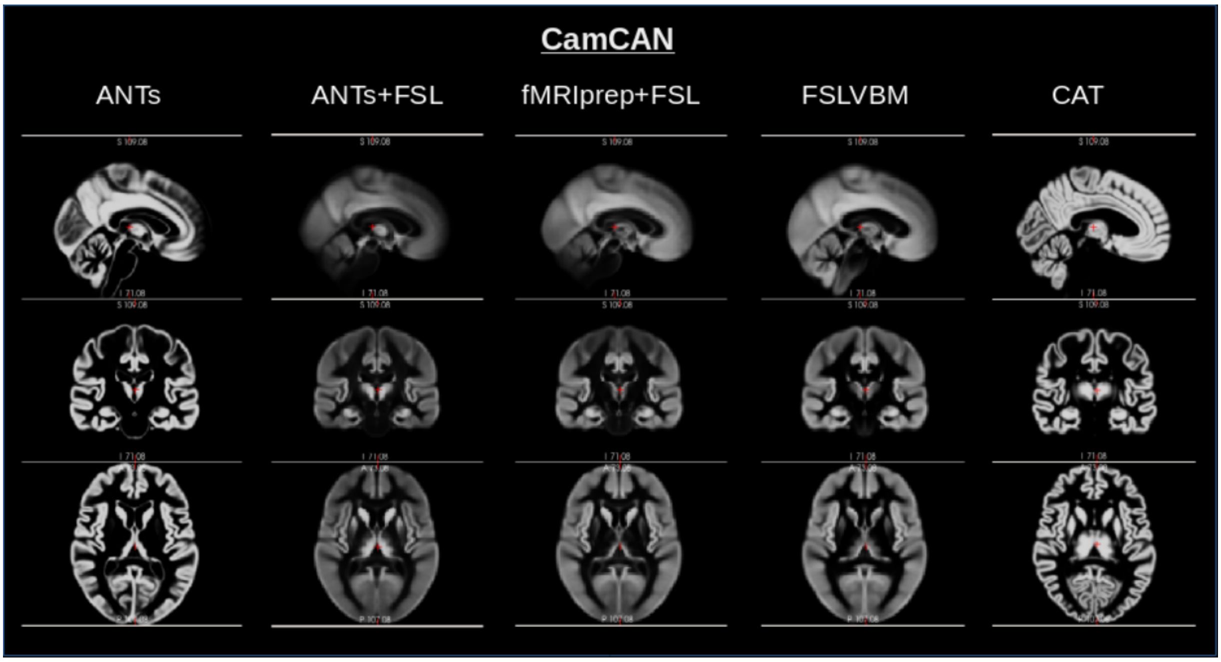

Figure S.2: Templates created for each pipeline CamCAN dataset.

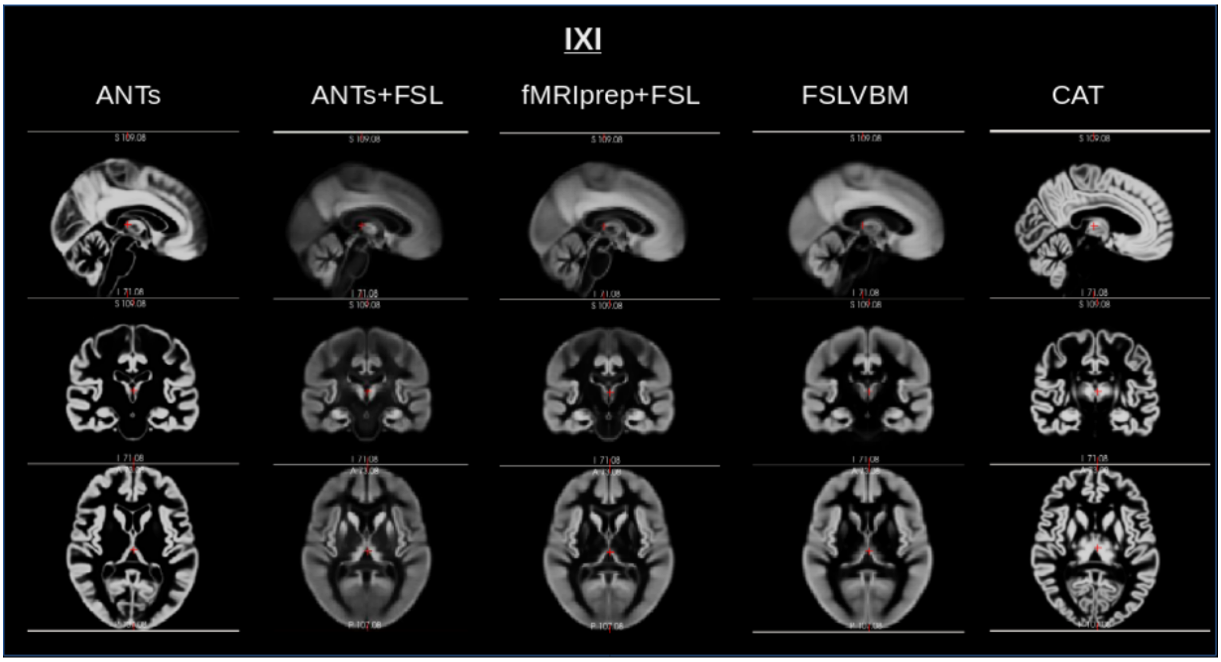

Figure S.3: Templates created for each pipeline for the IXI dataset

- Initialization of ANTs Atropos

It is worth mentioning that to follow our user-centric perspective, we used K-means clustering to initialize ANTs Atropos segmentation instead of some a-priori probability map. This resulted in relatively many subjects failing the preprocessing, as tissue samples were assigned unexpected labels by the K-means algorithm, resulting in confusion between white matter and gray matter. Although this could be approached by adjusting Atropos, such effort is out of scope of this project; instead, we performed custom quality control to detect such failed preprocessing.

- Detailed results of age predictions

Table S.2 shows the analytical results of age prediction when models are trained and tested within each site.

**Pipelines**	**Models**	**MAE per model all datasets**	**MAE all models all datasets**
ANTs General template	RVR	6.9	**7.06**
	GPR	7.04	
	LASSO	7.32	
	KRR	7.00	
ANTs Data template	RVR	6.93	**7.04**
	GPR	6.93	
	LASSO	7.35	
	KRR	6.95	
ANTs FSLVBM General template	RVR	6.56	**6.55**
	GPR	6.34	
	LASSO	6.79	
	KRR	6.52	
ANTs FSLVBM Data template	RVR	6.71	**6.74**
	GPR	6.46	
	LASSO	7.08	
	KRR	6.71	
fMRIPrep FSL General template	RVR	5.92	**5.83**
	GPR	5.65	
	LASSO	6.15	
	KRR	5.59	
fMRIPrep FSL Data template	RVR	6.14	**6.18**
	GPR	6.01	
	LASSO	6.51	
	KRR	6.06	
FSLVBM General template	RVR	6.25	**6.17**
	GPR	5.93	
	LASSO	6.45	
	KRR	6.05	
FSLVBM Data template	RVR	6.60	**6.55**
	GPR	6.30	
	LASSO	6.77	
	KRR	6.54	
CAT 12 General template	RVR	6.48	**6.39**
	GPR	6.26	
	LASSO	6.45	
	KRR	6.37	
CAT 12 Data template	RVR	6.46	**6.37**
	GPR	6.19	
	LASSO	6.47	
	KRR	6.37	

Table S.2: Results of age prediction using a multivariate approach. Four models were tested in the three datasets in a nested K-fold scheme. The third column contains the averaged results of the three datasets per model. The last column shows the average of all datasets and all models for each pipeline.

Table S.3 shows the analytical results of age prediction when the models are trained with

two of the datasets and tested with the leftout dataset.

Pipeline	Models	Test eNKI	Test CamCAN	Test IXI	Mean test (datasets)	Mean test (datasets & Pipelines)
ANTs General template	RVR	7.39	8.43	8.23	8.02	7.86
	GPR	7.19	7.94	8.27	7.80	
	LASSO	7.08	7.88	8.33	7.76	
	KRR	7.40	7.87	8.28	7.85	
ANTs Data template	RVR	7.48	8.26	7.88	7.87	7.86
	GPR	7.80	7.74	7.53	7.69	
	LASSO	7.73	6.88	8.99	7.87	
	KRR	7.84	8.12	8.03	8.00	
ANTs-FSL General template	RVR	6.96	9.16	9.75	8.62	8.44
	GPR	6.85	8.48	9.74	8.36	
	LASSO	7.60	7.97	9.88	8.49	
	KRR	6.73	7.91	10.28	8.31	
ANTs-FSL Data template	RVR	7.12	15.87	9.87	10.95	10.07
	GPR	6.95	11.47	9.72	9.38	
	LASSO	7.51	13.94	8.45	9.97	
	KRR	7.04	13.33	9.54	9.97	
FMRIprep-FSL General template	RVR	6.25	5.58	7.47	6.43	6.26
	GPR	6.10	5.63	7.13	6.29	
	LASSO	6.63	5.62	6.36	6.20	
	KRR	6.23	5.33	6.82	6.13	
FMRIprep-FSL Data template	RVR	6.83	6.56	6.12	6.50	6.21
	GPR	6.61	5.79	6.06	6.15	
	LASSO	6.66	5.76	5.91	6.11	
	KRR	6.48	5.89	5.82	6.06	
FSLVBM General template	RVR	6.62	6.02	8.66	7.10	6.77
	GPR	6.67	5.83	7.08	6.52	
	LASSO	7.23	6.09	6.88	6.73	
	KRR	6.43	5.77	8.00	6.73	
FSLVBM Data template	RVR	11.28	10.67	7.63	9.92	9.36
	GPR	11.42	10.66	6.70	9.55	
	LASSO	10.95	9.82	6.69	9.16	
	KRR	11.14	9.78	6.74	9.22	
CAT12.8 General template	RVR	6.75	6.53	6.39	6.55	6.45
	GPR	6.71	6.88	5.88	6.49	
	LASSO	6.75	6.62	5.92	6.43	
	KRR	6.55	6.58	5.82	6.32	
CAT12.8 Data template	RVR	6.83	6.81	6.25	6.63	6.76
	GPR	7.14	6.53	6.02	6.57	
	LASSO	7.20	7.51	7.52	7.41	
	KRR	7.01	6.06	6.19	6.42	

Table S.3: Cross-dataset age prediction results. For each pipeline we trained four models using two of the datasets and predicted the age of the subjects on the third, left-out dataset. The optimal parameters for each model were selected using a 5-fold cross validation scheme in the training datasets. The second to last column contains the mean of each model across all combinations of the datasets for training and testing for each pipeline. The last column contains mean across models and dataset combinations for each pipeline.

- Classifying subjects' images based on the preprocessing pipeline

We used two methods to scale the features prior to classification, using a linear SVM: i) within each feature and ii) within each subject, (both with standard scaling). The classification results were close to perfect using both methods (Figure S.4).

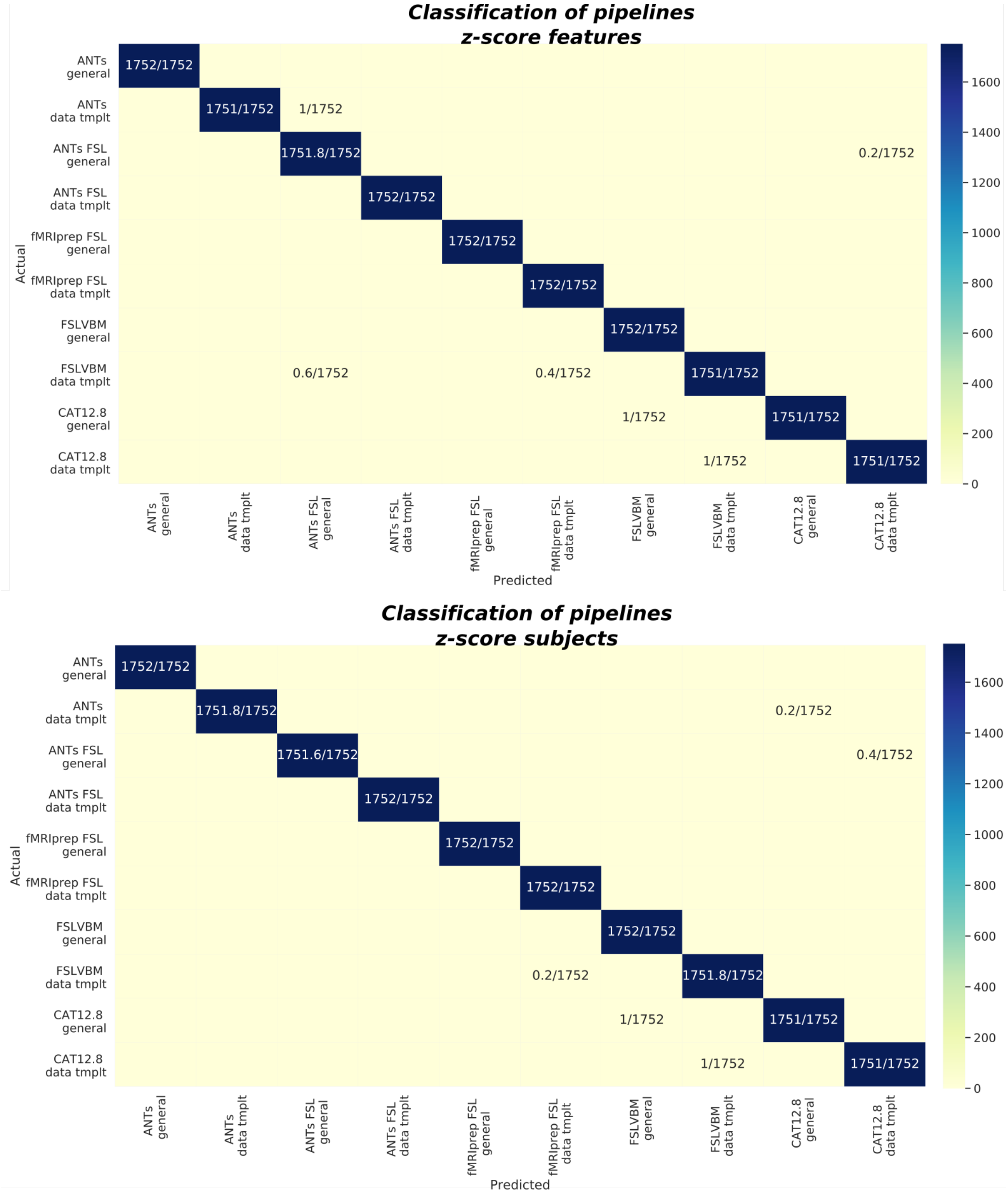

Figure S.4: Confusion matrices of multiclass classification of preprocessed subjects from all pipelines using the preprocessing pipelines as labels. We tried two methods for scaling the features aiming at ruling out that the overall intensity differences drive the classification. We used standard scaling, which standardizes features by removing the mean and scaling to unit variance, and MinMax scaling, which scales and translates each feature individually such that it is in the given range on the training set, here between zero and one.

- Harmonization across pipelines

The impact of harmonization across pipelines was also tested. We performed age-prediction and pipeline-prediction as we did for non-harmonized data in 2.4 and 2.5. Harmonization was performed using Neuroharmonize [1]. For age-prediction we selected eNKI processed by fMRIprep-FSL, IXI processed by FSLVBM and CamCAN processed by CAT 12.8 all with a general template. Table S.4 shows the age-prediction results when each of the three dataset is processed by different pipeline and then all data are harmonized.

model	Train: eNKI-IXI<br>Test: CamCAN	Train: eNKI-CamCAN<br>Test: IXI	Train: CamCAN-IXI<br>Test: eNKI	Average
RVR	MAE=10.7	MAE=8.11	MAE=7.9	MAE=8.9
GPR	MAE=10.3	MAE=7.6	MAE=7.5	MAE=8.5

Table S.4: Brain age prediction results for harmonized data. The brain age prediction process was performed using eNKI processed by fMRIprep-FSL, IXI processed by FSLVBM and CamCAN processed by CAT 12.8 all with a general template. Data were harmonized and then used for individuals age prediction with GPR and RVR as in 2.4.

The results of the pipeline classification with harmonized data are shown in Figure S.5.

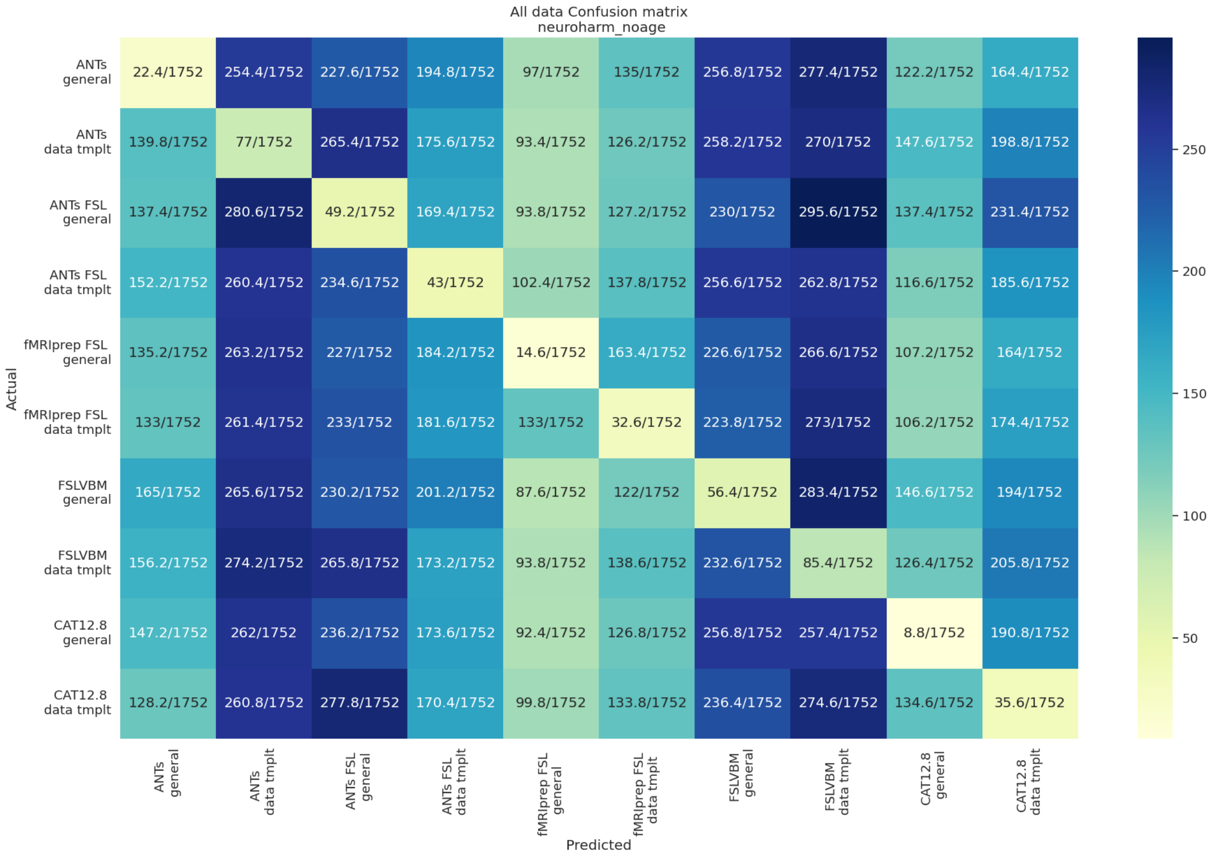

Figure S.5: Classification of pipelines based on harmonized GMV. Harmonization was performed across pipelines and the rest of the process was as in 2.5.

- Univariate analysis chart

Figure S.6 illustrates the univariate analysis we followed.

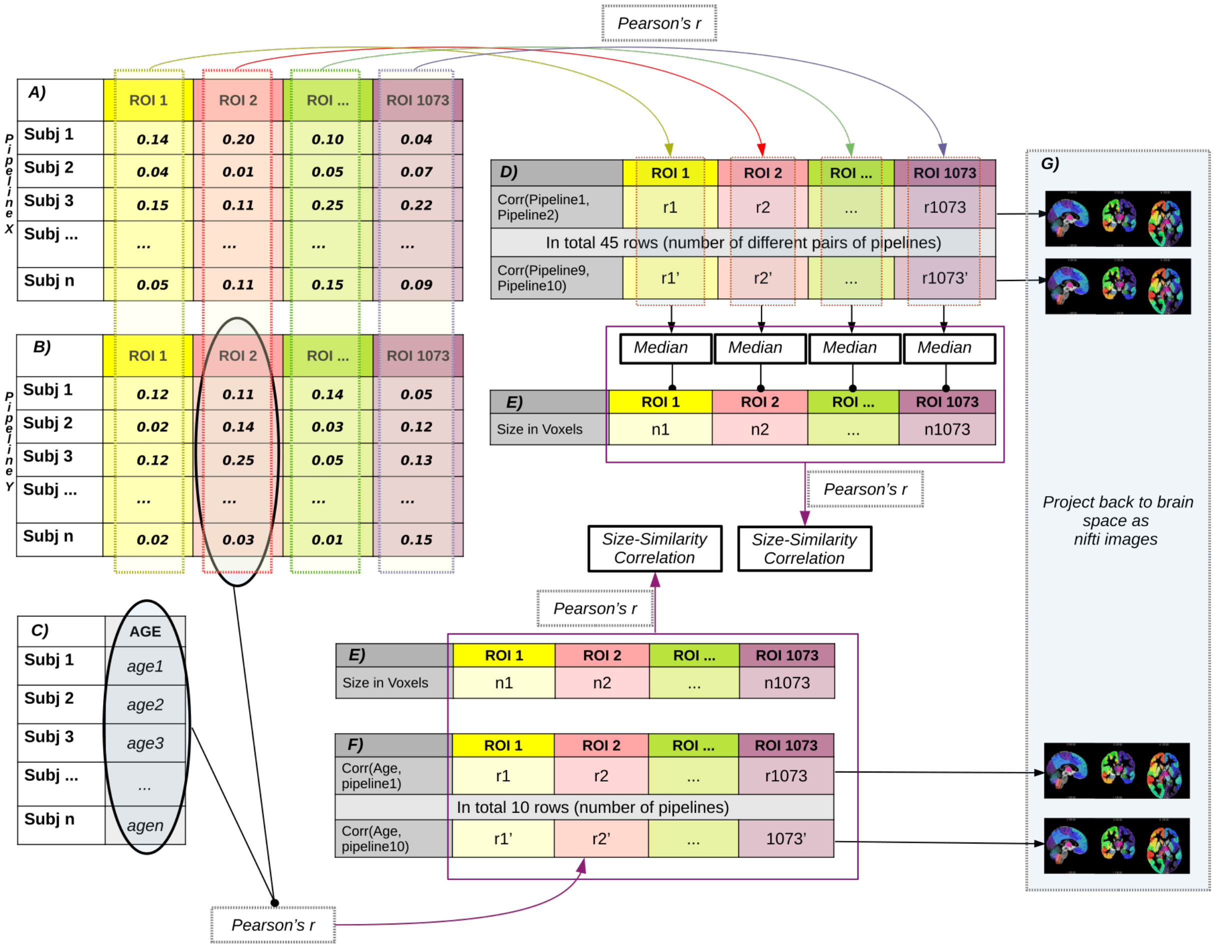

Figure S.6: Depiction of the process to calculate regional correlations across subjects for a pipeline pair. For a given pair of pipelines (panels A and B in the figure) for each region, we calculate the correlation across subjects. By performing this process for all pairs of pipelines and all regions, we obtain a regional correlation matrix (panel D). The overall agreement between the pipelines was calculated as the median for each region across all pipeline pairs, which was then used to correlate with the size of the parcels (panel E). For each pipeline and each region, we calculated Pearson's correlation across subjects between regional GMV (shown here for panel B) and age (panel C). Regional correlation values between pipelines (panel D) or with age (panel F) were projected on the brain for visualization purposes.

- Total GMV plots

The following image S.7 presents the total GMV of all subjects for each pipeline and each dataset.

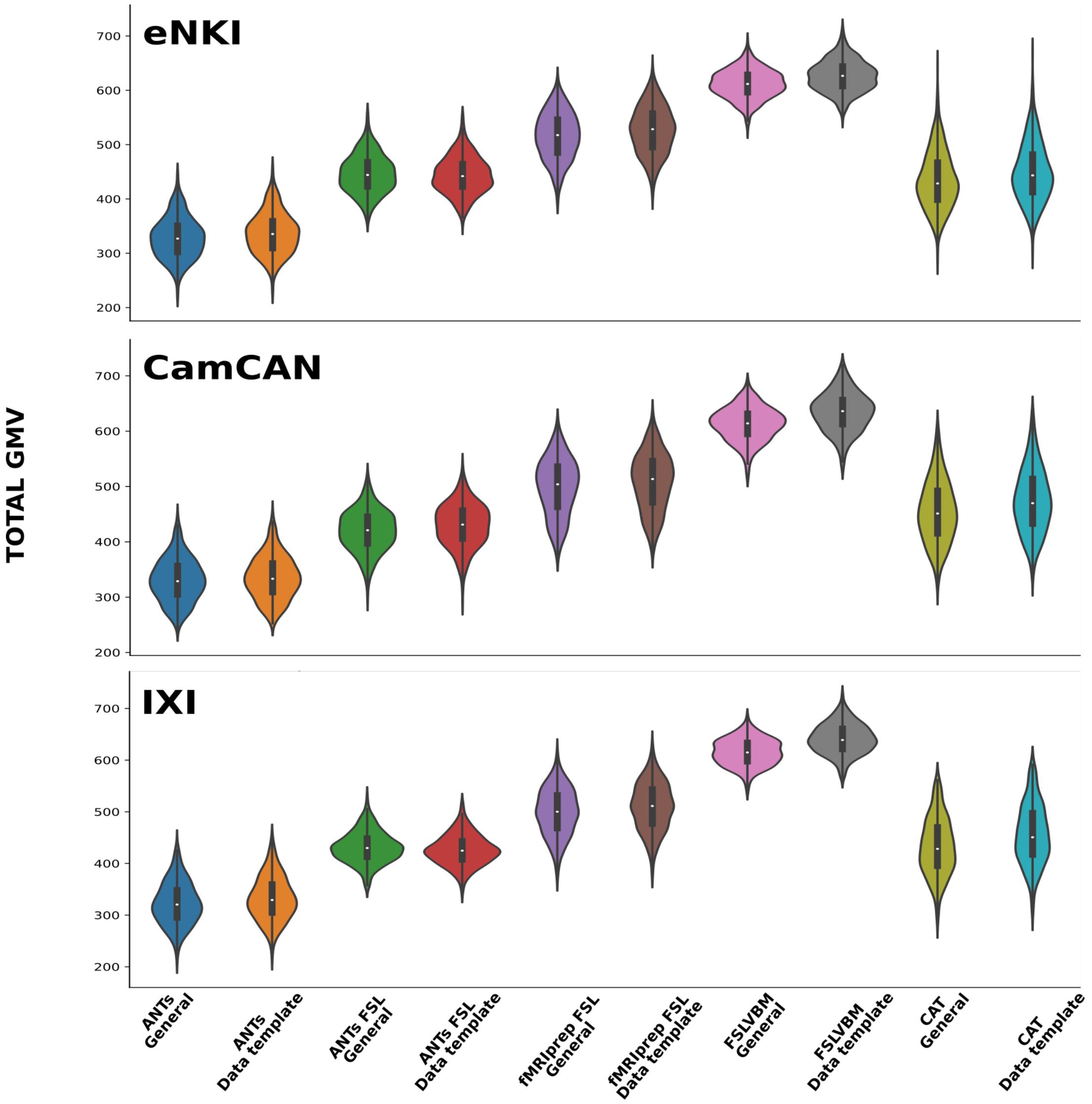

Figure S.7: Total GMV of all subjects for all pipelines and all datasets. Important differences in the total intensities between all pipelines. Only ANTs-FSL and CAT have similar means. Not surprisingly, the template appears to have no impact on the total GMV of subjects. High consistency is observed for the same pipelines across datasets.

- **Similarity between pipelines** as expressed by the regionwise Pearson's correlation across subjects for each pair of pipelines. For CamCAN in Figure S.9 and for IXI in S.10. From the figures of the three datasets, we see that similarities are consistent across datasets. However, some lack of variability is still identified, most likely due to differences in the quality of the images among datasets.

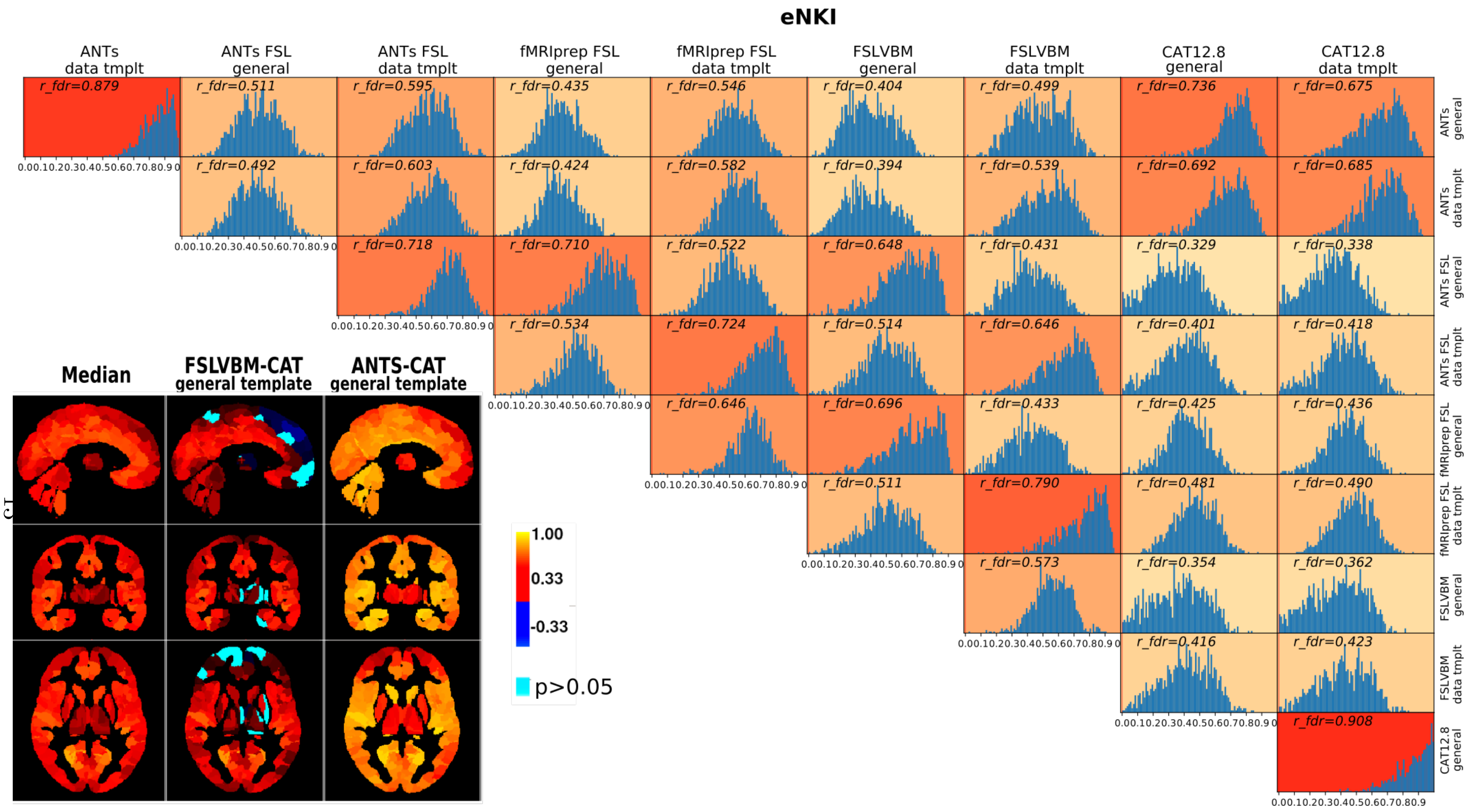

Figure S.8: eNKI: Histograms of Pearson's R values for all regions across subjects and for all combinations of pipelines. Niftis represent R values in the brain for the pipelines with Max mean, Min mean and the comparison of the two pipelines with the highest correlation to age.

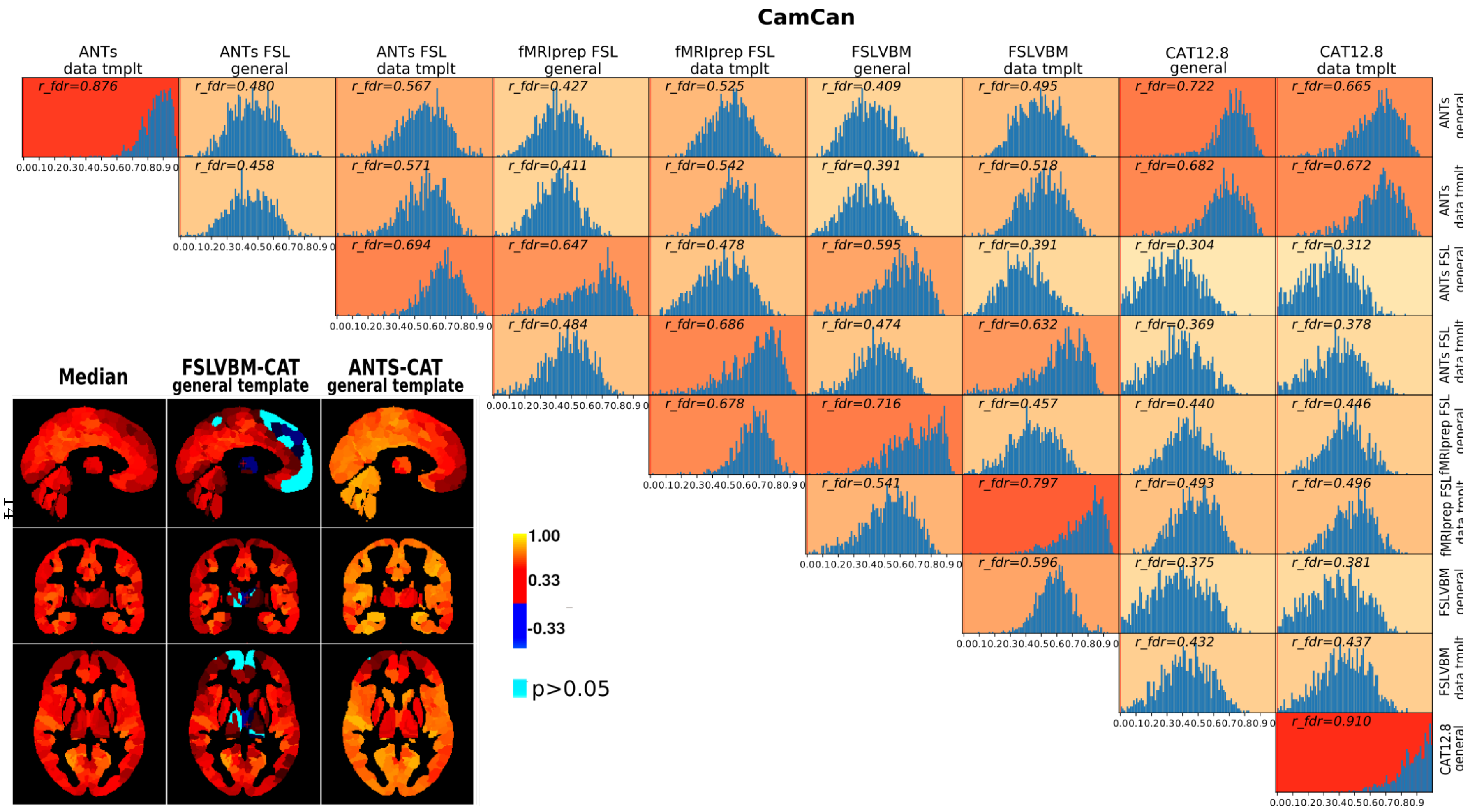

Figure S.9: CamCAN: Histograms of Pearson's R values for all regions across subjects and for all combinations of pipelines. Niftis represent R values in the brain for the pipelines with Max mean, Min mean and the comparison of the two pipelines with the highest correlation to age.

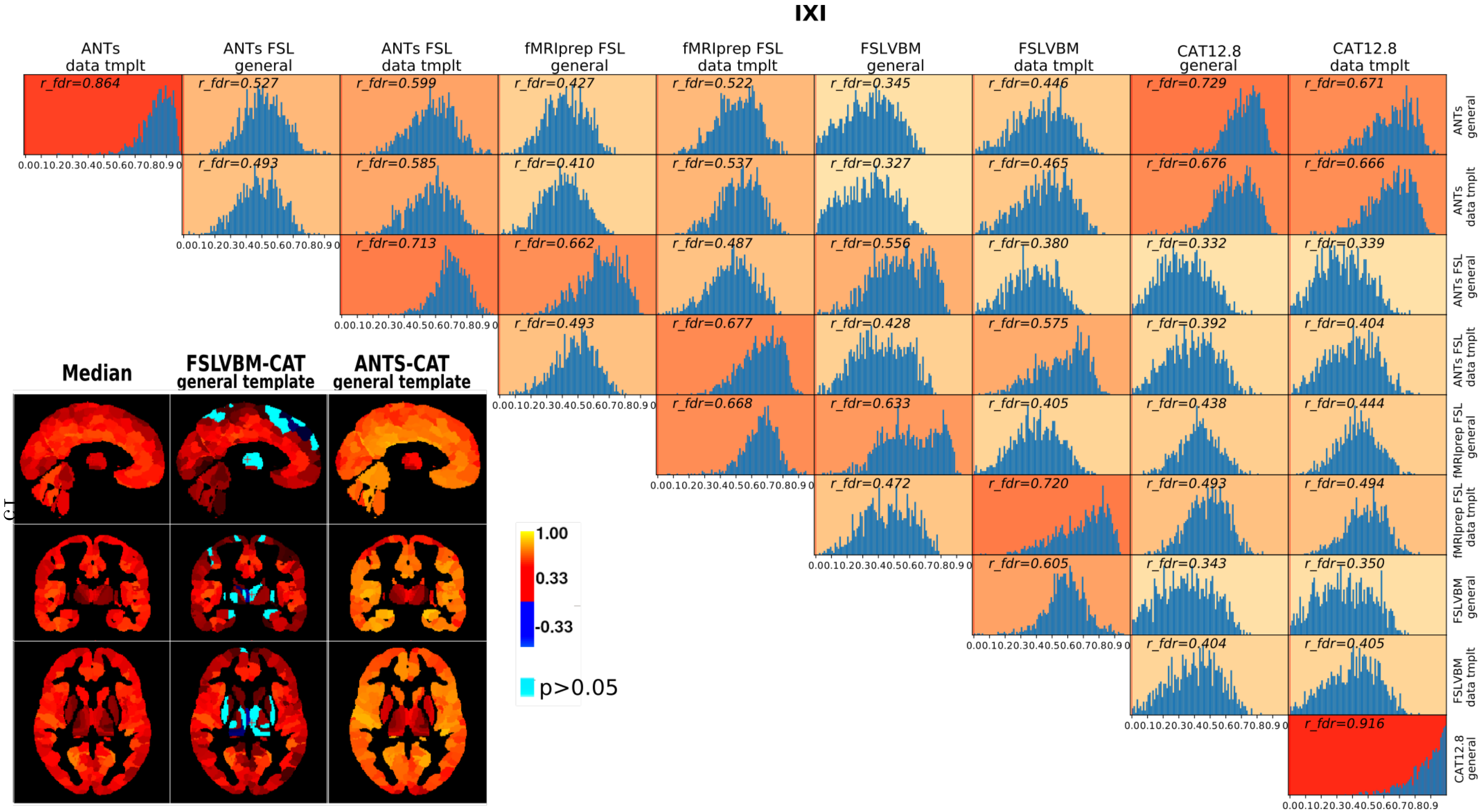

Figure S.10: IXI: Histograms of Pearson's R values for all regions across subjects and for all combinations of pipelines. Niftis represent R values in the brain for the pipelines with Max mean, Min mean and the comparison of the two pipelines with the highest correlation to age.

Correlations of pipelines that differ only in the template for the three datasets S.11

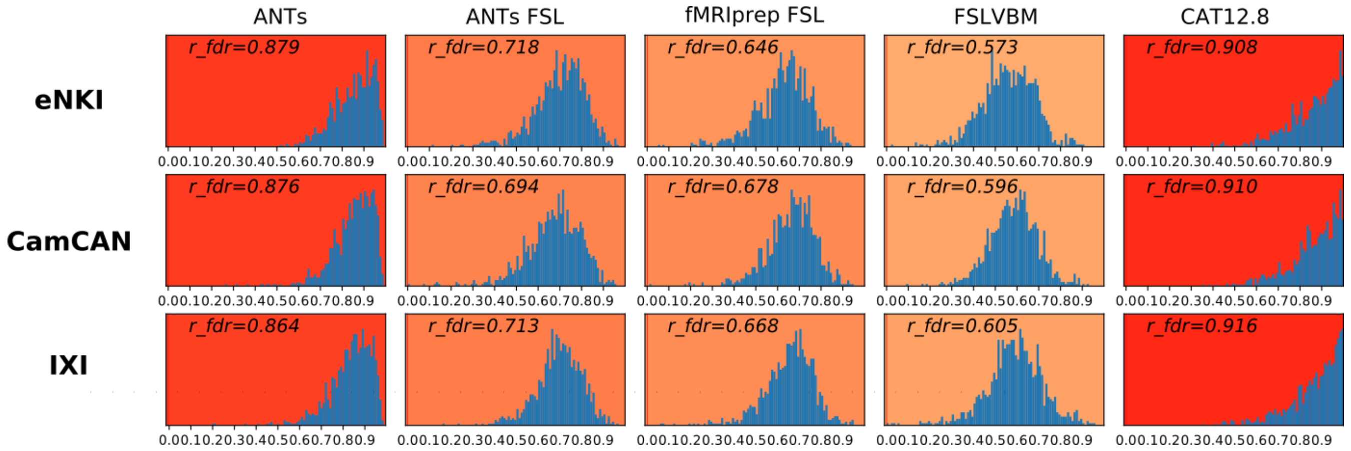

Figure S.11: Mean correlation of regions across subjects for all datasets between pipelines that only differ in the template used for spatial normalization.

The correlation between differential identifiability and Pearson's correlations calculated between pairs of pipelines was examined to assess the agreement between the two methods. The results can be seen in Figure S.12

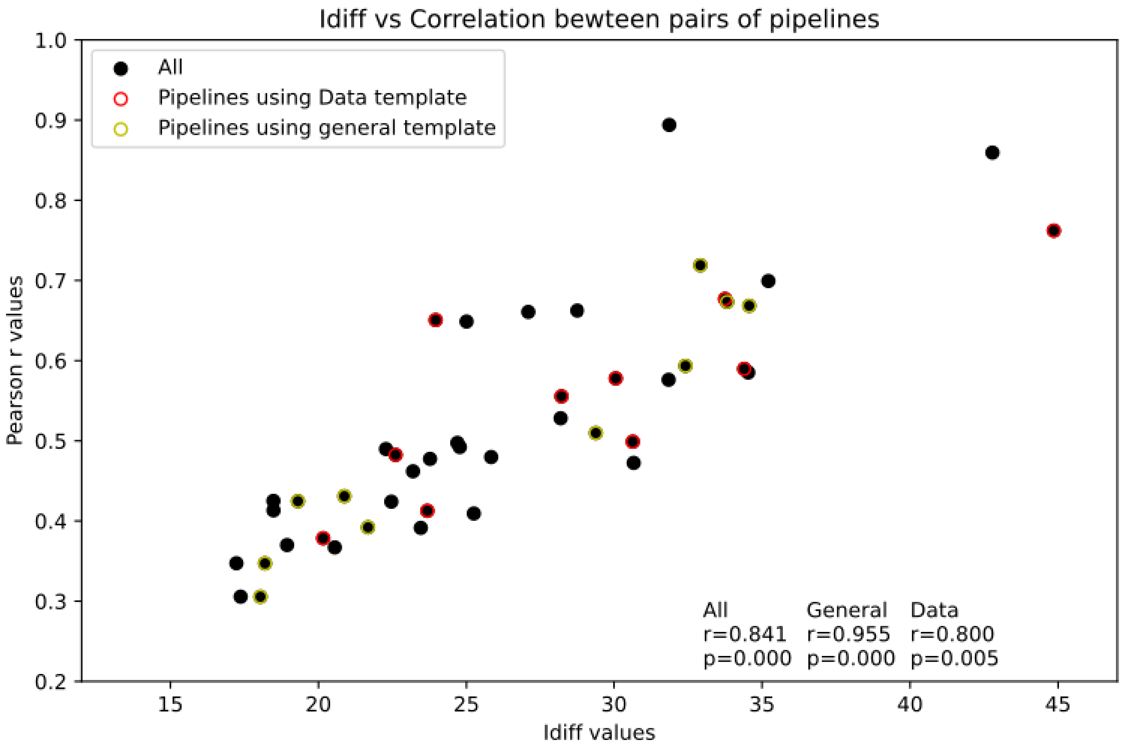

Figure S.12: Strong correlations were observed between the two methods we used to assess similarity between pipelines, the univariate analysis and identification. Especially between the pipelines using the general template, the correlation was $r = 0.955$. For pipelines using data-templates, the correlation was $r = 0.8$. The correlation for all pipeline pairs was $r = 0.841$. All correlations had $p < 0.05$.

Age-ROIs correlations for all pipelines in Figure S.13

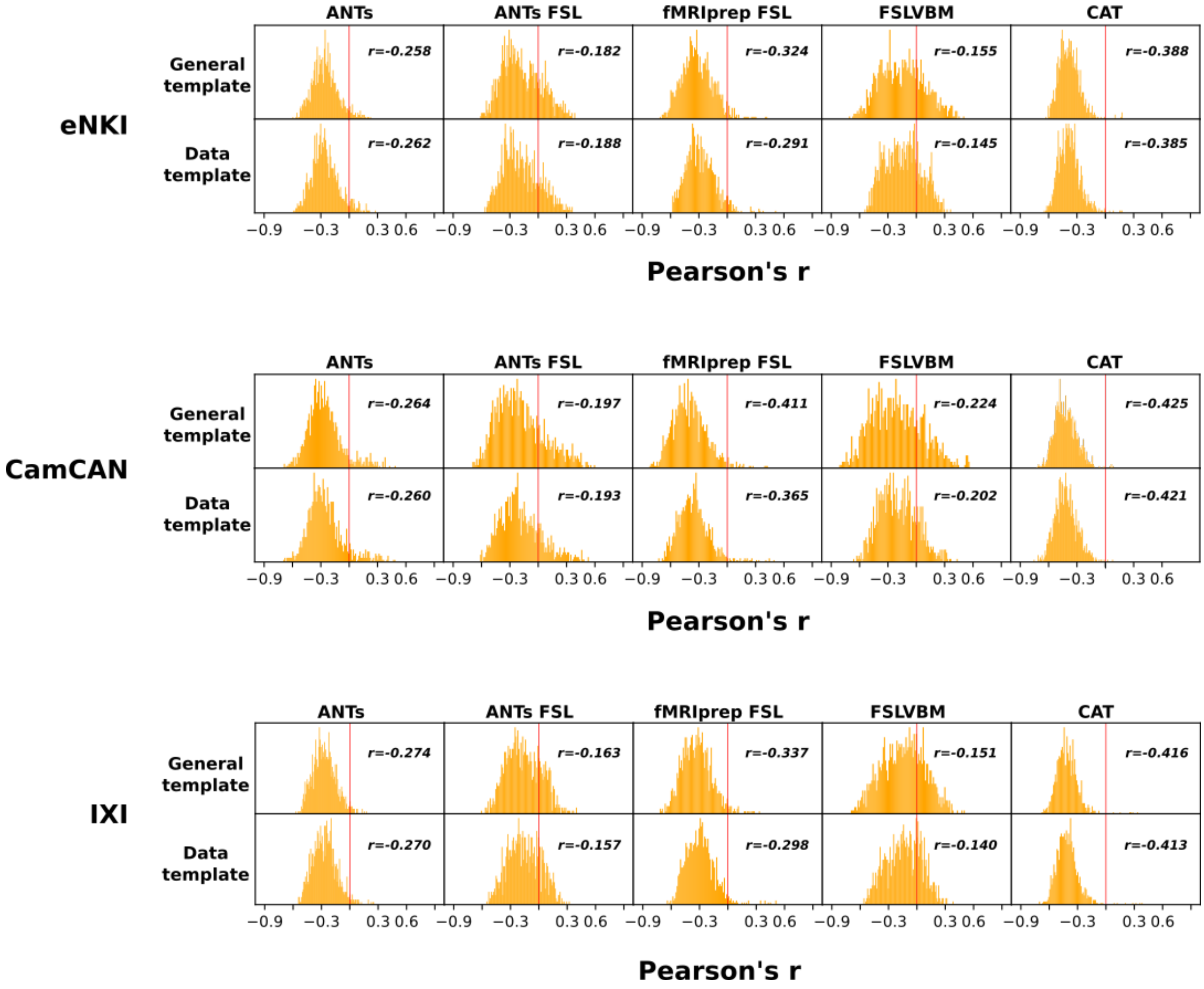

Figure S.13: Correlation between regions and age of subjects for all pipelines.

Table S.5 ANOVA results for correlation values between ROIs and age for all pipelines across subjects of all datasets. Figure S.14 depicts the same correlation values between all regions and age calculated across subjects of all datasets, per pipeline.

Dataset	F score	p-value
**eNKI**	509.99	4.18E-193 <0.05
**CamCAN**	400.45	5.11E-156 <0.05
**IXI**	637.771	5.18E-234 <0.05
**All data**	324.468	5.18E-234 <0.05

Table S.5: One-way ANOVA for the three datasets was performed for the three pipelines that used general templates and had the highest overall correlation to age.

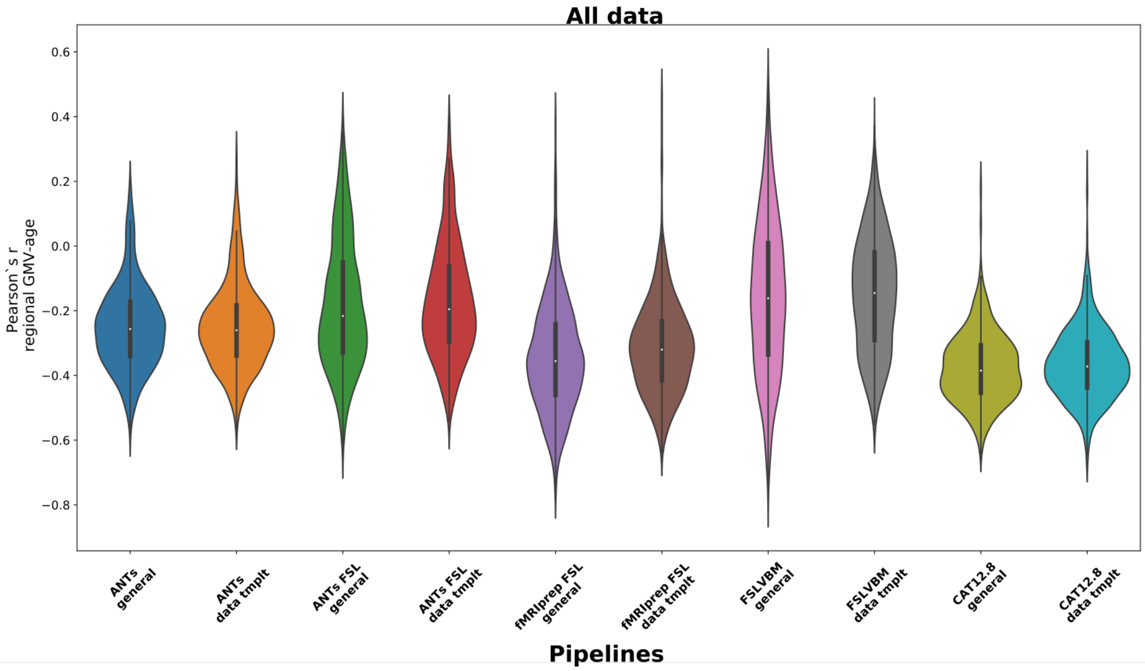

Figure S.14: ROI-age correlations for all pipelines and all datasets. One-way ANOVA showed that there were significant differences between pipelines in ROI-Age correlations.

Scatter plots for each pipeline demonstrate the size of each ROI on the x-axis and the Pearson's r value between ROI and age calculated across subjects. The first figure (Figure S.21) is for the CamCAN dataset, and Figure S.22 is for IXI.

Paired comparisons of ROI-Age correlation values between pipelines

Figure S.15 shows pairplots of age-region correlations across subjects for all data, and Figures S.16, S.17 and S.18 show for eNKI, CamCAM and IXI, respectively.

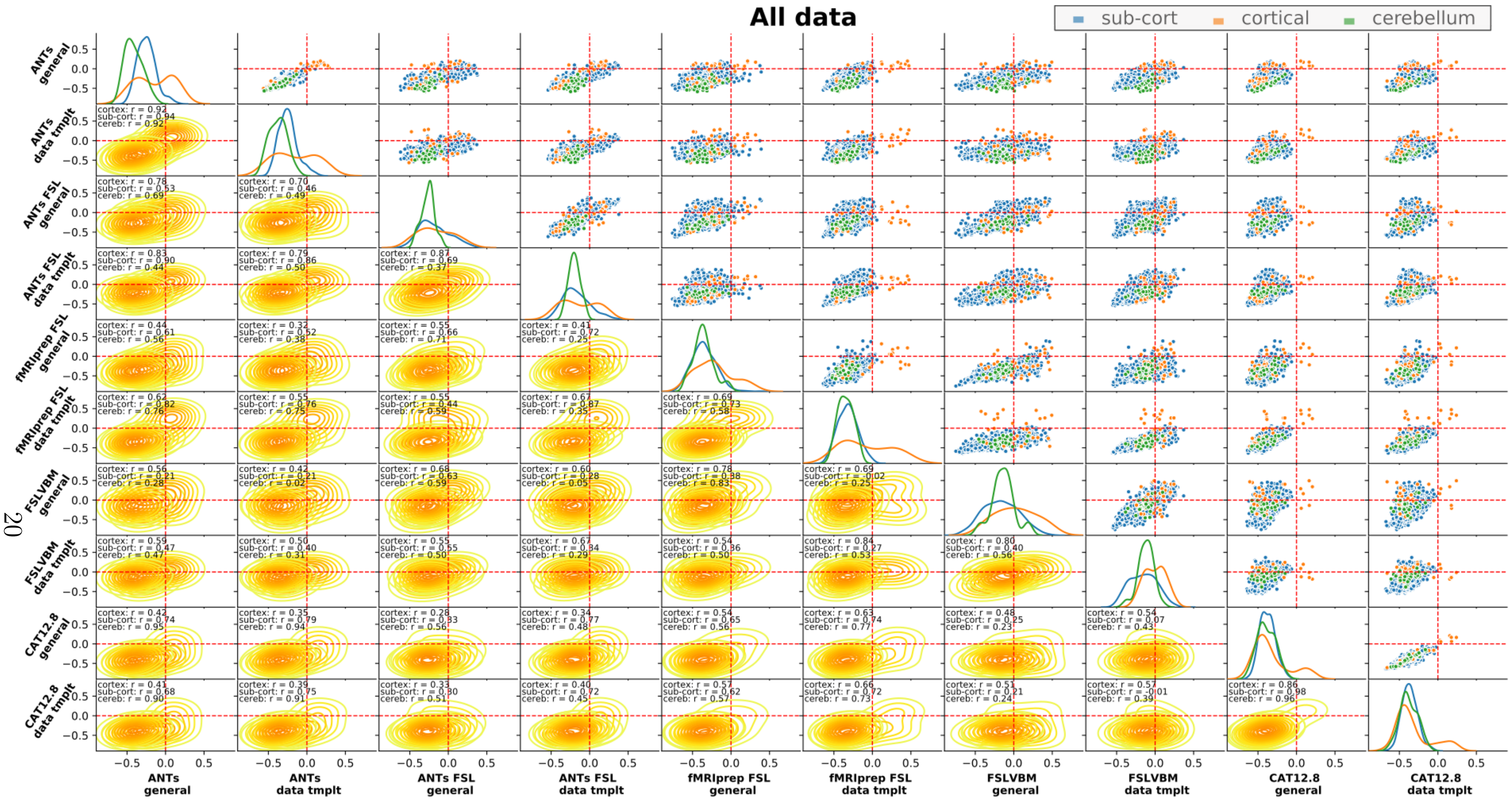
All data
sub-cort
cortical
cerebellum
ANTs general
ANTs data tmplt
ANTs FSL general
ANTs FSL data tmplt
fMRIprep FSL general
fMRIprep FSL data tmplt
FSLVBM general
FSLVBM data tmplt
CAT12.8 general
CAT12.8 data tmplt
cortex: r = 0.92 sub-cort: r = 0.94 cereb: r = 0.92
cortex: r = 0.78 sub-cort: r = 0.53 cereb: r = 0.69
cortex: r = 0.70 sub-cort: r = 0.46 cereb: r = 0.49
cortex: r = 0.83 sub-cort: r = 0.90 cereb: r = 0.44
cortex: r = 0.79 sub-cort: r = 0.86 cereb: r = 0.50
cortex: r = 0.87 sub-cort: r = 0.69 cereb: r = 0.37
cortex: r = 0.44 sub-cort: r = 0.61 cereb: r = 0.56
cortex: r = 0.32 sub-cort: r = 0.52 cereb: r = 0.38
cortex: r = 0.55 sub-cort: r = 0.66 cereb: r = 0.71
cortex: r = 0.41 sub-cort: r = 0.72 cereb: r = 0.25
cortex: r = 0.62 sub-cort: r = 0.82 cereb: r = 0.76
cortex: r = 0.55 sub-cort: r = 0.76 cereb: r = 0.75
cortex: r = 0.55 sub-cort: r = 0.44 cereb: r = 0.59
cortex: r = 0.67 sub-cort: r = 0.87 cereb: r = 0.35
cortex: r = 0.69 sub-cort: r = 0.73 cereb: r = 0.58
cortex: r = 0.56 sub-cort: r = 0.21 cereb: r = 0.28
cortex: r = 0.42 sub-cort: r = 0.21 cereb: r = 0.02
cortex: r = 0.68 sub-cort: r = 0.63 cereb: r = 0.59
cortex: r = 0.60 sub-cort: r = 0.28 cereb: r = 0.05
cortex: r = 0.78 sub-cort: r = 0.88 cereb: r = 0.83
cortex: r = 0.69 sub-cort: r = -0.02 cereb: r = 0.25
cortex: r = 0.59 sub-cort: r = 0.47 cereb: r = 0.47
cortex: r = 0.50 sub-cort: r = 0.40 cereb: r = 0.31
cortex: r = 0.55 sub-cort: r = 0.55 cereb: r = 0.50
cortex: r = 0.67 sub-cort: r = 0.34 cereb: r = 0.29
cortex: r = 0.54 sub-cort: r = 0.36 cereb: r = 0.50
cortex: r = 0.84 sub-cort: r = 0.27 cereb: r = 0.53
cortex: r = 0.80 sub-cort: r = 0.40 cereb: r = 0.56
cortex: r = 0.42 sub-cort: r = 0.74 cereb: r = 0.95
cortex: r = 0.35 sub-cort: r = 0.79 cereb: r = 0.94
cortex: r = 0.28 sub-cort: r = 0.33 cereb: r = 0.56
cortex: r = 0.34 sub-cort: r = 0.77 cereb: r = 0.48
cortex: r = 0.54 sub-cort: r = 0.65 cereb: r = 0.56
cortex: r = 0.63 sub-cort: r = 0.74 cereb: r = 0.77
cortex: r = 0.48 sub-cort: r = 0.25 cereb: r = 0.23
cortex: r = 0.54 sub-cort: r = 0.07 cereb: r = 0.43
cortex: r = 0.41 sub-cort: r = 0.68 cereb: r = 0.90
cortex: r = 0.39 sub-cort: r = 0.75 cereb: r = 0.91
cortex: r = 0.33 sub-cort: r = 0.30 cereb: r = 0.51
cortex: r = 0.40 sub-cort: r = 0.72 cereb: r = 0.45
cortex: r = 0.57 sub-cort: r = 0.62 cereb: r = 0.57
cortex: r = 0.66 sub-cort: r = 0.72 cereb: r = 0.73
cortex: r = 0.51 sub-cort: r = 0.21 cereb: r = 0.24
cortex: r = 0.57 sub-cort: r = -0.01 cereb: r = 0.39
cortex: r = 0.86 sub-cort: r = 0.98 cereb: r = 0.96
-0.5 0.0 0.5

Figure S.15:

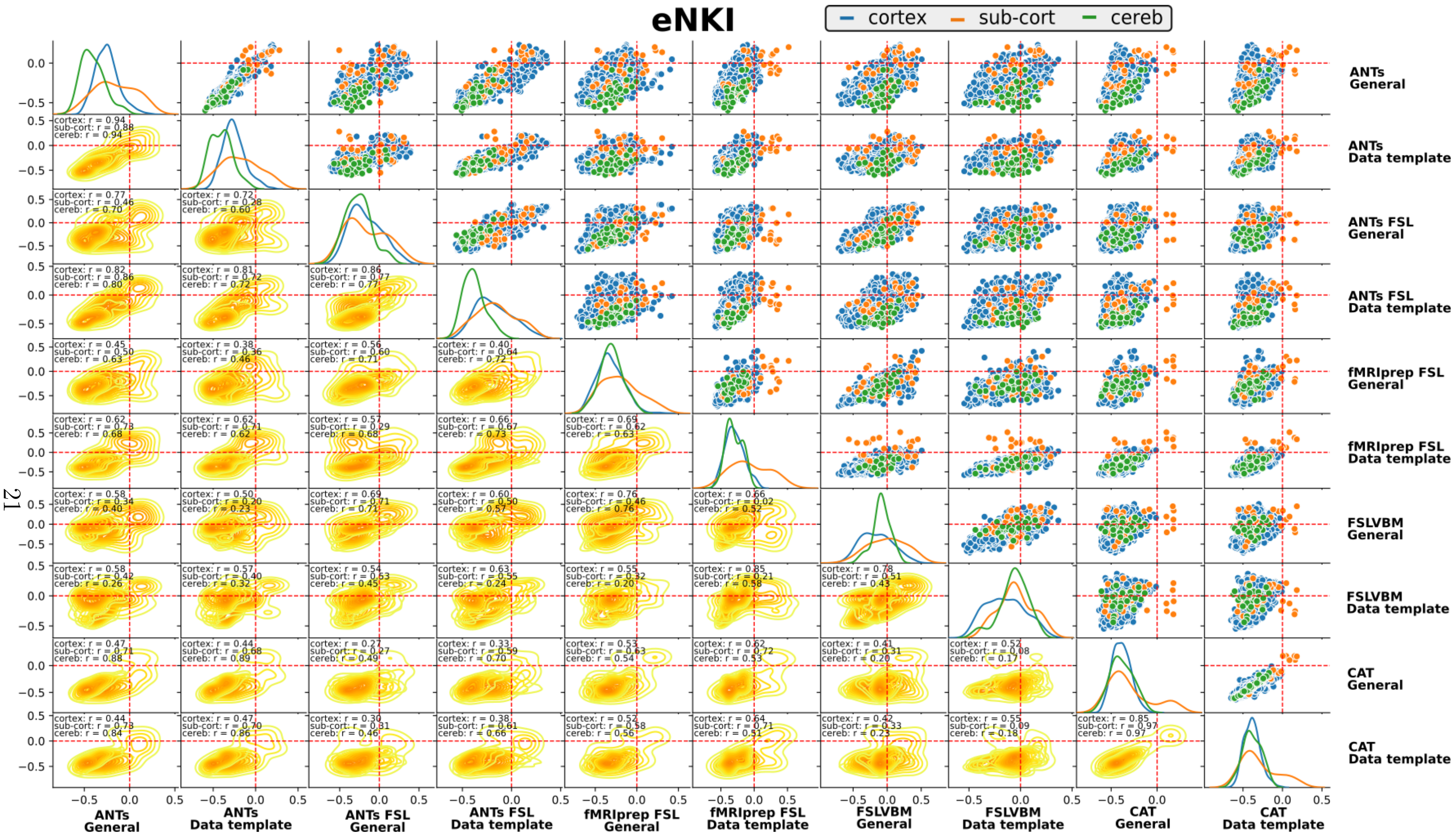
eNKI
cortex
sub-cort
cereb
ANTs General
ANTs Data template
ANTs FSL General
ANTs FSL Data template
fMRIprep FSL General
fMRIprep FSL Data template
FSLVBM General
FSLVBM Data template
CAT General
CAT Data template

Figure S.16:

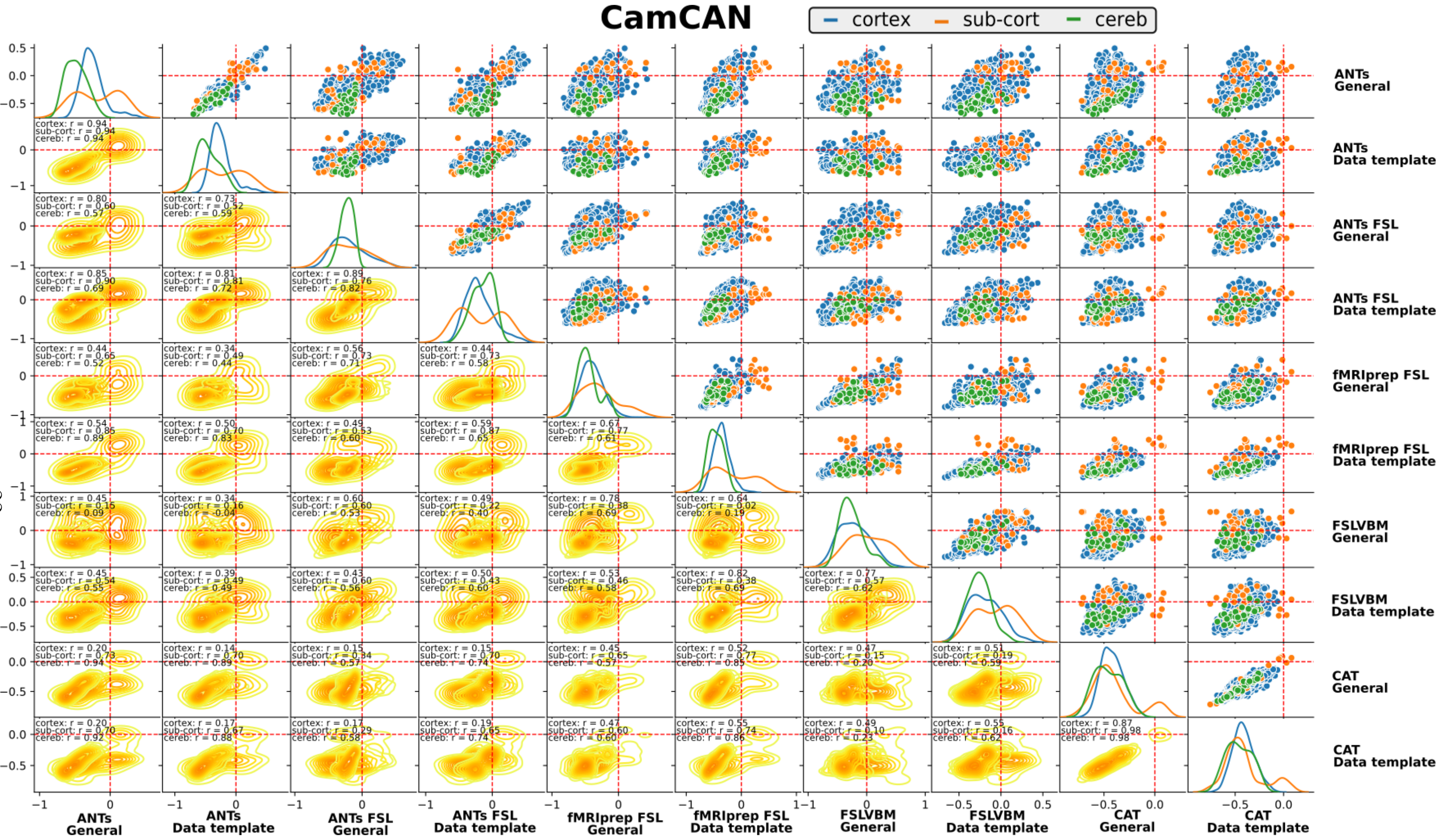
CamCAN
cortex
sub-cort
cereb
ANTs General
ANTs Data template
ANTs FSL General
ANTs FSL Data template
fMRIprep FSL General
fMRIprep FSL Data template
FSLVBM General
FSLVBM Data template
CAT General
CAT Data template

Figure S.17:

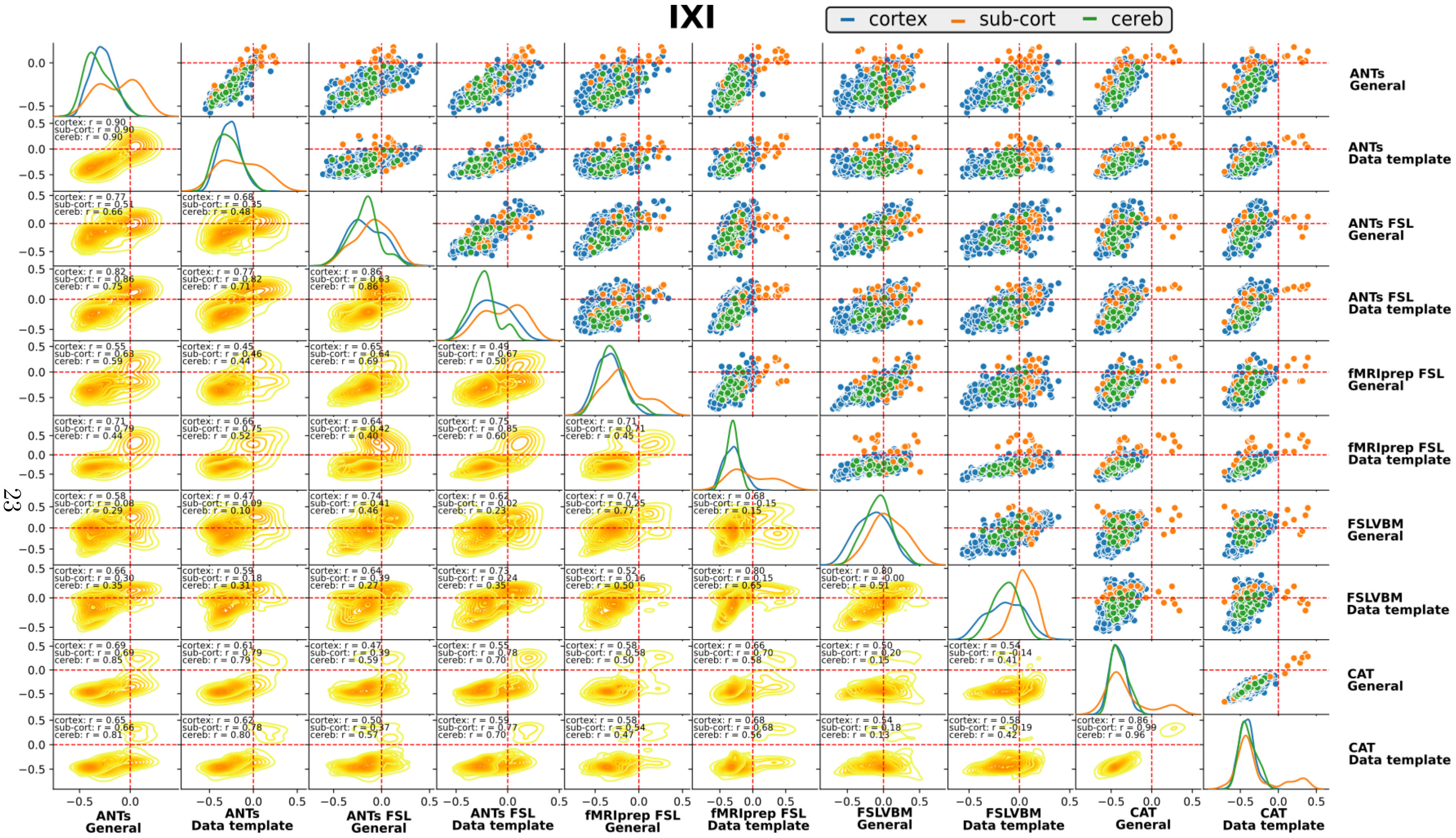

IXI
cortex
sub-cort
cereb
ANTs General
ANTs Data template
ANTs FSL General
ANTs FSL Data template
fMRIprep FSL General
fMRIprep FSL Data template
FSLVBM General
FSLVBM Data template
CAT General
CAT Data template

Figure S.18:

The effect of region size

- Association between the overall similarity among the pipelines (calculated as the median of agreement between pairs) and parcel sizes

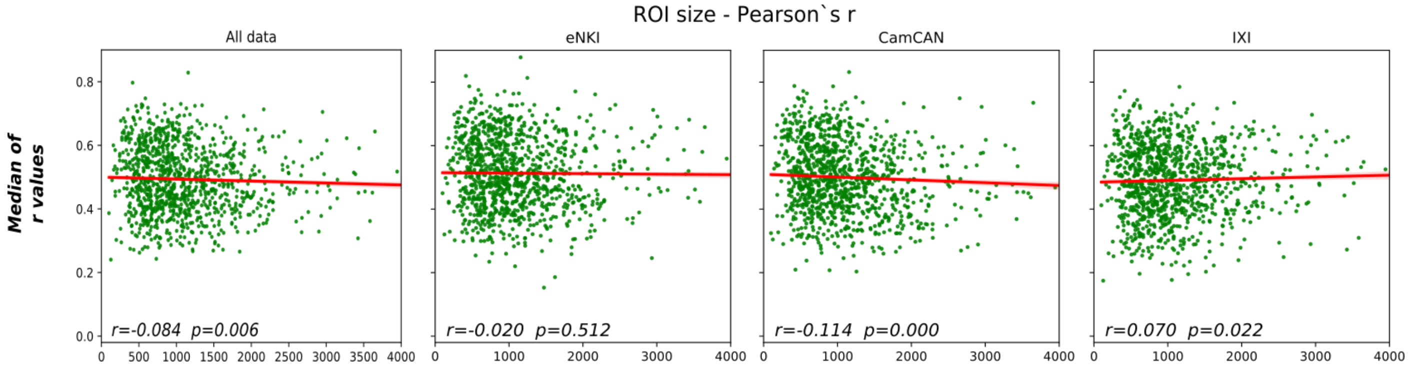

Figure S.19: Median values from all pairs of pipelines of Pearson's r correlations across subjects for all regions plotted against the size of the regions. A nonsignificant correlation was found for the eNKI dataset, and very low correlations were found for the other two datasets.

-The association between the size of regions and the corresponding ROI-age correlation values. CAT appears to have a higher association between the size of each ROI and the correspondence correlation value with age for eNKI (r=-0.128 for both templates) and Cam-CAN. ANTs had similar values but only for the eNKI dataset (=r=-0.121 for general template and -0.125 for data template). For the IXI dataset, FSLVBM with the general template had the highest values (r=0.102). Notably, FSLVBM had a positive correlation when ANTs and CAT had negative values. Figure S.23 provides the same analysis when data from all the datasets are combined.

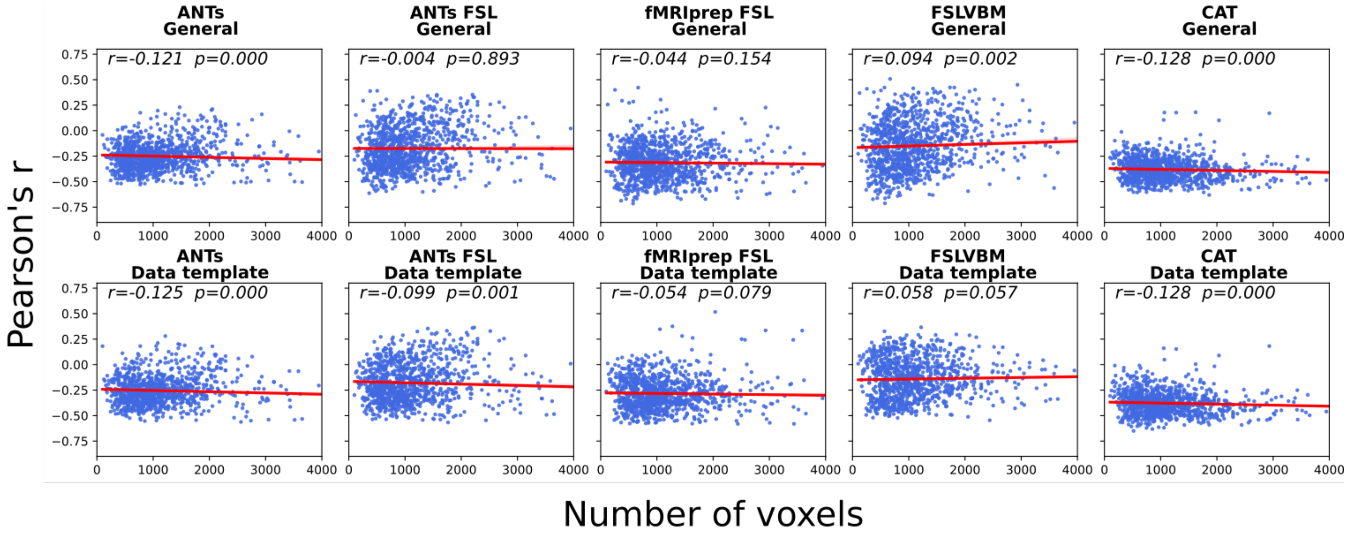

Figure S.20: Scatter plots with the y-axis representing regional correlation to age and the x-axis representing the size of the corresponding ROI, for all pipelines estimated in all datasets. For each pipeline, we estimated the Pearson's r and p values. Red lines represent the linear regression line.

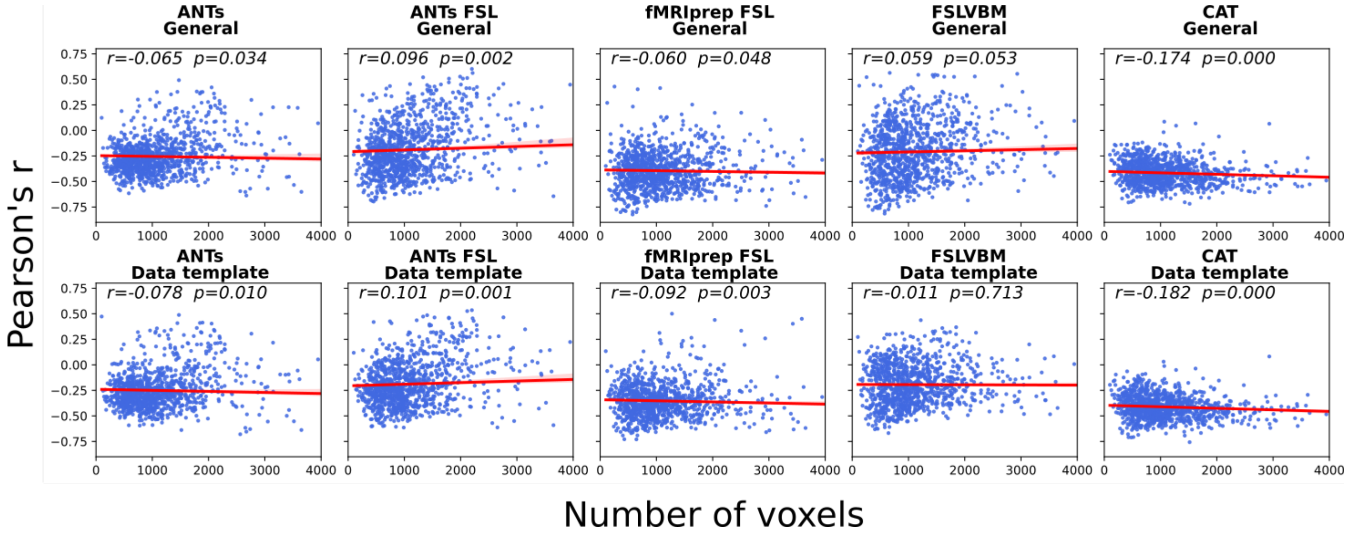

Figure S.21: Scatter plots with the y-axis representing regional correlation to age and the x-axis representing the size of the corresponding ROI for all pipelines estimated in the eNKI dataset. For each pipeline, we estimated the Pearson's r and p values. Red lines represent the linear regression line.

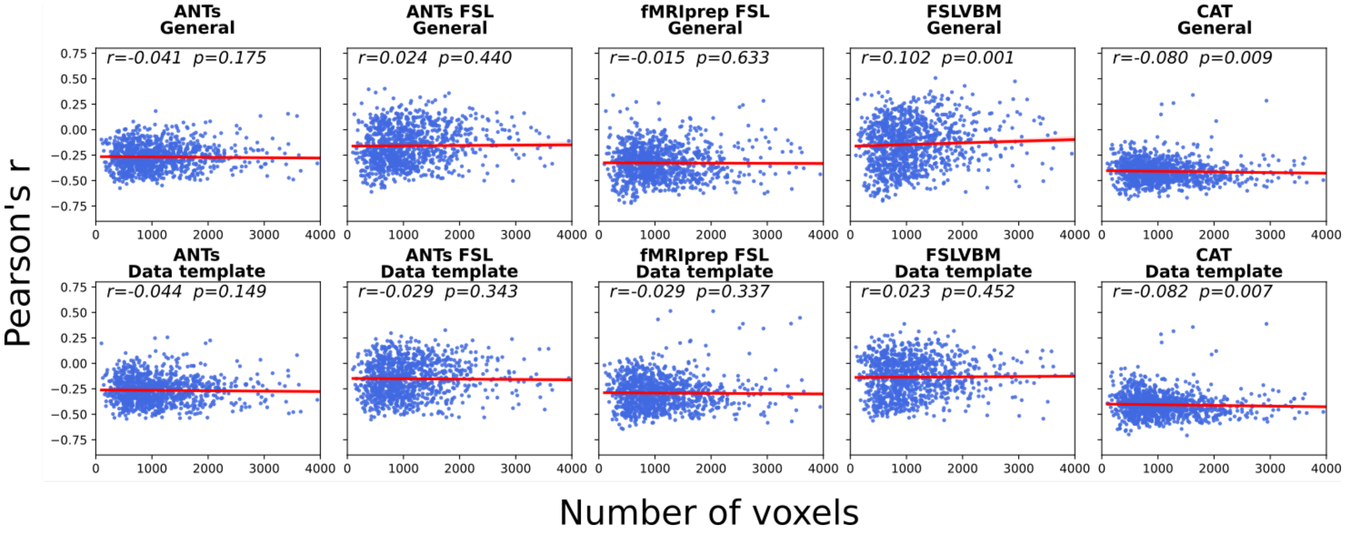

Figure S.22: Scatter plots with the y-axis representing regional correlation to age and the x-axis representing the size of the corresponding ROI for all pipelines estimated in the Cam-CAN dataset. For each pipeline, we estimated the Pearson's r and p values. Red lines represent the linear regression line.

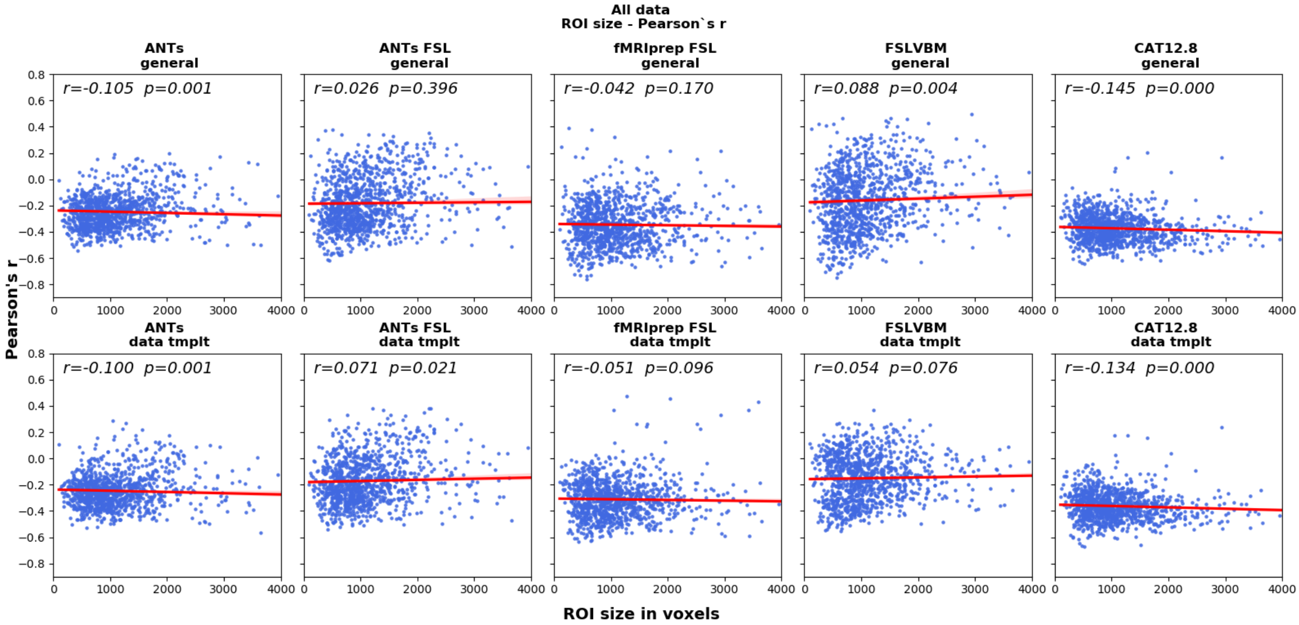

Figure S.23: Scatter plots with the y-axis representing regional correlation to age and the x-axis representing the size of the corresponding ROI for all pipelines estimated in the IXI dataset. For each pipeline, we estimated the Pearson's r and p values. Red lines represent the linear regression line.

[1] Garcia-Dias, Rafael, Scarpazza, Cristina, Baecker, Lea, Vieira, Sandra, Pinaya, Walter H.L., Corvin, Aiden, Redolfi, Alberto, Nelson, Barnaby, Crespo-Facorro, Bene-dicto, Mc-Donald, Colm, Tordesillas-Gutiérrez, Diana, Cannon, Dara, Mothersill, David, Hernaus, Dennis, Morris, Derek, Setien-Suero, Esther, Donohoe, Gary,Frisoni, Giovanni, Tronchin, Giulia, Sato, João, Marcelis, Machteld, Kempton, Matthew, van Haren, Neeltje E.M., Gru-ber, Oliver, McGorry, Patrick, Amminger, Paul, McGuire, Philip, Gong, Qiyong, Kahn, René S., Ayesa-Arriola, Rosa,van Amelsvoort, Therese, Ortiz-García de la Foz, Victor, Calhoun, Vince,Cahn, Wiepke, Mechelli, Andrea, 2020. Neuroharmony: A new tool for harmonizing volumetric MRI data from unseen scanners. Neuroimage 220, 117127.
